# Effects of Number of Repetitions and Number of Hours of Shaping Practice during Constraint-Induced Movement Therapy: A Randomized Controlled Trial

**DOI:** 10.1155/2018/5496408

**Published:** 2018-04-02

**Authors:** Auwal Abdullahi

**Affiliations:** Department of Physiotherapy, Bayero University Kano, Kano, Kano State, Nigeria

## Abstract

**Background:**

Constraint-induced movement therapy (CIMT) is effective in improving motor outcomes after stroke. However, its existing protocols are resource-intensive and difficult to implement. The aim of this study is to design an easier CIMT protocol using number of repetitions of shaping practice.

**Method:**

The study design was randomized controlled trial. Participants within 4 weeks after stroke were recruited at Murtala Muhammad Specialist Hospital. They were randomly assigned to groups A, B, C, and D. Group A received 3 hours of traditional therapy. Groups B, C, and D received modified CIMT consisting of 3 hours of shaping practice per session, 300 repetitions of shaping practice in 3 sessions, and 600 repetitions of shaping practice in 3 sessions per day, respectively, and constraint for 90% of the waking hours. All treatment protocols were administered 5 times per week for 4 weeks. The primary outcome was measured using upper limb Fugl-Meyer assessment, while the secondary outcome was measured using motor activity log, Wolf Motor Function Test, and upper limb self-efficacy test at baseline, 2 weeks, and 4 weeks after intervention.

**Result:**

There were 48 participants 4 weeks after intervention. The result showed that there was no significant difference between groups at baseline (*p* > 0.05). Within-group improvements attained minimal clinically important difference (MCID) in modified CIMT and 300 repetitions and 600 repetitions groups.

**Conclusion:**

Number of repetitions of shaping practice significantly improved motor function, real-world arm use, and upper limb self-efficacy after stroke. Therefore, it seems to be a simple alternative for the use of number of hours.

**Trial Registration:**

This trial is registered with Pan African Clinical Trial Registry (registration number: PACTR201610001828172) (date of registration: 21/10/2016).

## 1. Background

The acute stage after stroke is significant, since it offers the opportunity for spontaneous recovery and responds well to rehabilitation, and the biologic window period is within it. Thus, if well utilized, it is a period where disability due to stroke may be prevented or significantly reduced. Reduction of disability is the goal number three of the Sustainable Development Goals (SDGs) [1].

To prevent or significantly reduce disability after stroke, constraint-induced movement therapy (CIMT) is used [2, 3]. The effectiveness of CIMT is demonstrated in improving various outcomes after stroke such as laboratory and real-world arm use and changes in the brain activity and metabolism and movement precision, and quality has been reported in the literature [3–6]. However, in the existing protocols of CIMT, patients may have to spend between 0.5 and 6 hours per day practicing task or shaping coupled with wearing a constraint for several hours to 95% of the waking hours. These kinds of protocols seem to be resource-intensive [7] and may be difficult to adopt where there are constraints in the availability of staff and service affordability by the patients. Additionally, some studies have reported that the number of hours claimed during CIMT was not completely used for task practice [8–10]. Thus, it is difficult to know how much task or shaping the patients practiced when number of hours is used as a component of dose of task practice.

One of the main components of CIMT is repetitive tasks/shaping practice. Studies in neurorehabilitation have shown a linear relationship between number of repetitions and recovery of motor function [11–14]. One of the studies in particular indicated that when tasks were practiced about 300 times per day for 2 weeks, the participants had significant improvement in motor function and that the 300 repetitions were possible within just one hour [13]. This finding may have implication for practice, since it seems to suggest that it is not the number of hours spent practicing task but rather the number of repetitions of the task that is important for motor recovery. Thus, knowing the exact number of repetitions our patients need for recovery of motor function can be an important rehabilitation milestone. However, these studies lack external validity as they are either a case report or a noncontrolled trial. Therefore, the aim of this study is to rigorously compare CIMT protocols using number of repetitions of shaping practice with the one using number of hours of shaping practice using a randomized controlled trial (RCT) design.

## 2. Method

### 2.1. Study Design

The study was an RCT comparing the use of number of repetitions of shaping practice and number of hours of shaping practice during CIMT. The study protocol was explained in detail previously [15].

### 2.2. Participants

The study participants were consecutive stroke patients who were not more than 4 weeks after stroke, with no very severe impairment in motor function as indicated by a score of 1 to 3 on the motor arm item of the National Institutes of Health Stroke Scale (NIHSS) and a score of 3 or more on the upper arm item of the Motor Assessment Scale (MAS). Additionally, the study participants had no severe cognitive impairment as indicated by a score of 1 or less on the consciousness and communication items of the NIHSS, ability to perform two-step commands, and a score of less than 8 on the Short Blessed Memory Orientation and Concentration Scale (SBMOCS) [16–18]. Furthermore, the study participants had no upper extremity injury that led to activity limitation before the stroke. However, participants were excluded if they had more than 3 errors on the Star Cancellation Test and sensory loss of 2 or more on the sensory item of NIHSS [18].

The sample size (*n* = 73) was determined using G∗Power software version 3.1 [19]. However, 3 was added to make it 76 so that the groups will have equal number of participants. The effect size and power and alpha values used for the calculation of the sample size were 0.4 (large effect size), 0.05, and 0.8, respectively. That means the type of power analysis is a priori. The outcome used for the calculation of the sample size is upper limb Fugl-Meyer (FM) which was reported to have clinically important difference value between 4.25 and 7.25 points [20].

Participants' recruitment has been carried out by a trained therapist at Murtala Muhammad Specialist Hospital from 6 February 2017 to date. Since the participants are being recruited consecutively, simple random sampling was used to randomize the participants into the study groups. Four therapists who were blinded to the aim of the study were randomly selected using folded opaque papers marked with letters A to D. The therapists represent 1 group each. Thereafter, a study assistant wrote numbers representing the study sample size on pieces of opaque papers, folded them, and mixed them for several times in a small bowl. He then asked each therapist representing a particular group to pick equivalent amount of the pieces of papers. Therefore, the recruitment number as the patients come consecutively determines the participant's study group.

### 2.3. Ethical Consideration

The study was approved by the Ethics Subcommittee of Operational Research Advisory Committee of Kano State Ministry of Health. The approval number is MOH/Off/797/T.I/176. The protocol of the study was also registered with Pan African Clinical Trial Registry (registration number: PACTR201610001828172), which is available at http://www.pactr.org.

### 2.4. Procedure

The study protocol was explained in detail to the participants and their caregivers, and the informed consents of the participants were obtained. However, the participants were blinded to what each other did, and they were requested not to discuss their treatment with any other participants in order to avoid contamination effect.

### 2.5. Intervention and Control

Group A (the control) received 3 hours of traditional therapy consisting of passive movement, therapeutic positioning, and weight bearing on the affected limb. Group B (the mCIMT) received modified CIMT consisting of 3 hours of shaping practice per session per day and constraint for 90% of the waking hours. Group C received 300 repetitions of shaping practice in 3 sessions per day (100 repetitions per session) and constraint for 90% of the waking hours. Group D received 600 repetitions of shaping practice in 3 sessions per day (200 repetitions per session) and constraint for 90% of the waking hours. The shaping practice performed included picking up a cup from the table, taking it to the mouth, and drinking from it, writing letters or drawing a circle, transferring an object (cell phone) from left to right on a table, taking the hand from the lap to the head and sliding it from the front to the back, brushing the teeth, taking the hand to the nose, and putting and removing shoes. The shaping practice entails tasks being broken down into manageable components according to the patient's motor ability at the time and with progression as the motor ability of the patient improves. Each of these 5 tasks was carried out 20 and 40 times per session in groups C and D, respectively, while they were carried out for 3 hours in group B.

The rationale for choosing 300 and 600 repetitions is that, in the animal and human literature, tasks repetition in the range of 300 to 800 repetitions was reported to be required for motor learning [11–14]. All treatment protocols were administered 5 times per week for 4 weeks (on the first day and on weekly visit) under the supervision of a trained therapist (who was blinded to the groups the participants belong to) and informal caregivers (who supervised the participants at home). Further observation and assessment by the therapist and assessor occurred on a weekly basis. Occasional telephone calls (every 3 days) and a log book (daily record) were used to monitor compliance with the protocol.

### 2.6. Study Outcomes

The study outcomes include motor function, perceived motor function, motor impairment, and upper limb self-efficacy. The primary outcome was measured using motor function subscale of upper limb Fugl-Meyer (FM) assessment, while the secondary outcomes were measured using WMFT, motor activity log, and upper limb self-efficacy test (UPSET). The upper limb Fugl-Meyer motor function subscale assesses motor function and coordination/speed of the extremity, hand, and wrist following stroke [21]. The scale is scored from 0 to 2, where 0 = cannot perform, 1 = performs partially, and 2 = performs fully. Its scores range from a minimum of 0 to a maximum of 66. The instrument is reported to be stable [22] and has excellent internal consistency [23]. The Wolf Motor Function Test (WMFT) is a time-based measure of single or multiple joints' motions and functional tasks of the upper limb [24]. It consists of 17 items that are scored on a scale from 0 to 5 (with 0–5 indicating increasing ability). The measure has been reported to have good construct and criterion-related validity and interrater reliability [25]. The motor activity log (MAL) consists of 2 subscales that measure amount of use and quality of movement with 30 items in total assessing the use of the affected hand in daily tasks [26]. Each item is scored on a scale from 0 to 5 points with 0 to 5 indicating increasing perceived motor function. The scale has been reported to be valid and reliable [27, 28]. The UPSET is a measure of how confident the patient is in the use of his upper limb in carrying out daily activities. It has been shown to correlate well with Stroke Self-efficacy Questionnaire and has good internal consistency [29]. It consists of 20 items that are scored on a scale from 0 to 10 (with 0 to 10 indicating increasing confidence). All measurements were performed at baseline, 2 weeks, and 4 weeks after intervention by a blinded assessor. The therapist who assessed the participants for eligibility was the one who sent the participants to the outcomes assessor. In this way, the assessor was blinded.

### 2.7. Data Analysis

The demographic data was analyzed using descriptive statistics. The data for the study outcomes obtained was assessed for normality using Kolmogorov-Smirnov statistics. After that, the differences in the study outcomes between groups at baseline were analyzed using one-way between-groups ANOVA and time-group interaction in the study outcomes was analyzed using mixed within-between-groups ANOVA.

## 3. Result

**Figure 1:**
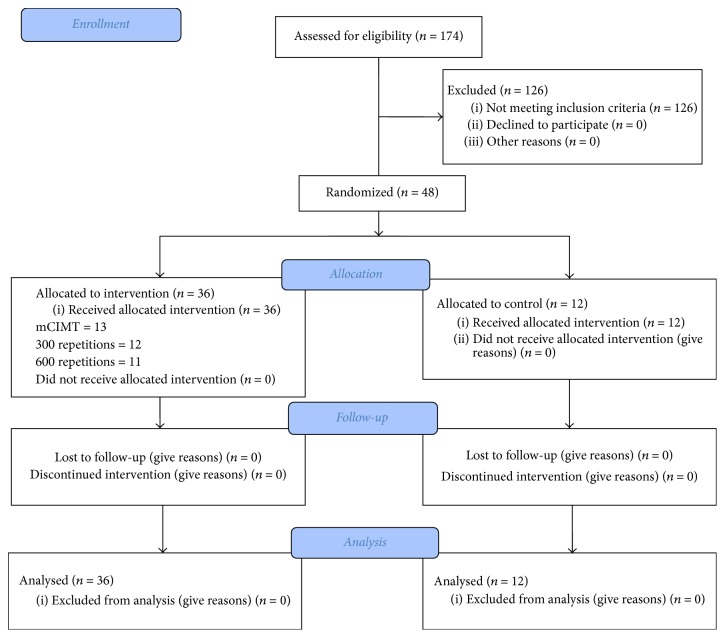
The study's flow diagram.

Between 20 February 2017 and 29 September 2017, 48 participants were enrolled in the study and the data on the outcome of interests at baseline and at 2 and 4 weeks after intervention were collected. The study's flow chart is represented in Figure 1.

**Figure 2:**
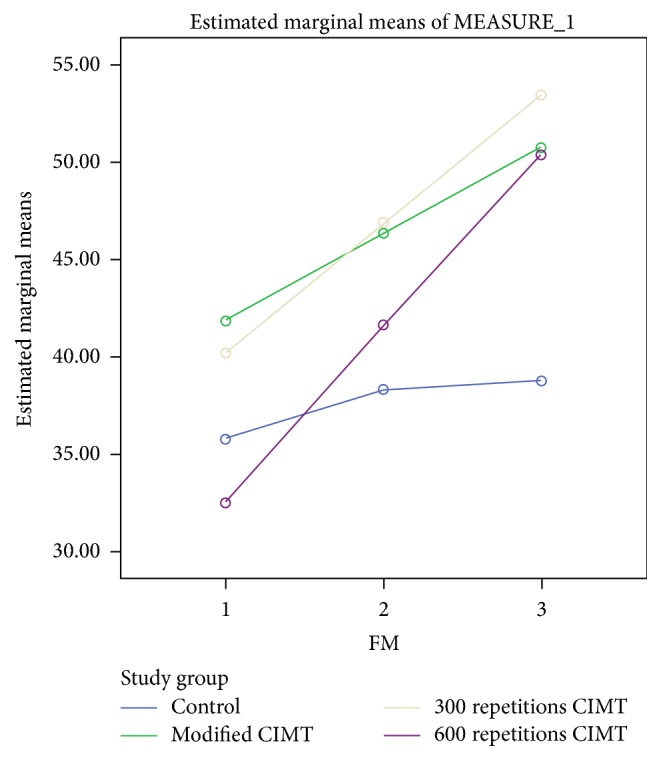
A profile plot showing groups' mean scores in FM at baseline, 2 weeks, and 4 weeks after intervention.

The baseline characteristics of the study participants including the baseline scores in the outcomes of interest are presented in Table 1. The protocols of the study were well adhered as reported by the relatives of the study participants using the log books and by the therapist that administered treatment on the first day and monitored compliance during weekly visits. The results showed that there were no significant differences between groups (*p* > 0.05) in the study outcomes at baseline. For the study outcomes, a mixed within-between-groups ANOVA was conducted to assess the effect of four different interventions (control, mCIMT, 300 repetitions, and 600 repetitions) on FM, MAL how well, MAL amount of use, WMFT, and UPSET. For FM, there was significant interaction between group and time, Wilks' lambda = .612, *F*(3, 48) = 3.903, *p* = 0.002, and partial *η*^2^ = 0.218. This shows that the changes in FM scores over time were not the same between groups. The improvement in FM scores attained minimal clinically important difference (MCID) between baseline and 2 weeks and between 2 weeks and 4 weeks after intervention in only mCIMT, 300 repetitions, and 600 repetitions groups (see Table 2 and Figure 2). The MCID for upper limb Fugl-Meyer (FM) ranges from 4.25 to 7.25 points [20]. However, there was a substantial main effect for time, Wilks' lambda = 0.23, *F*(3, 48) = 62.058, *p* < 0.001, and partial *η*^2^ = 0.747, with all groups showing increase in FM scores across the three time periods (see Figure 2). The main effect comparing the 3 groups was not significant (*F*(3, 48) = 1.662, *p* = 0.189, and partial *η*^2^ = 0.104), suggesting no difference in the effectiveness of the four interventions.

**Figure 3:**
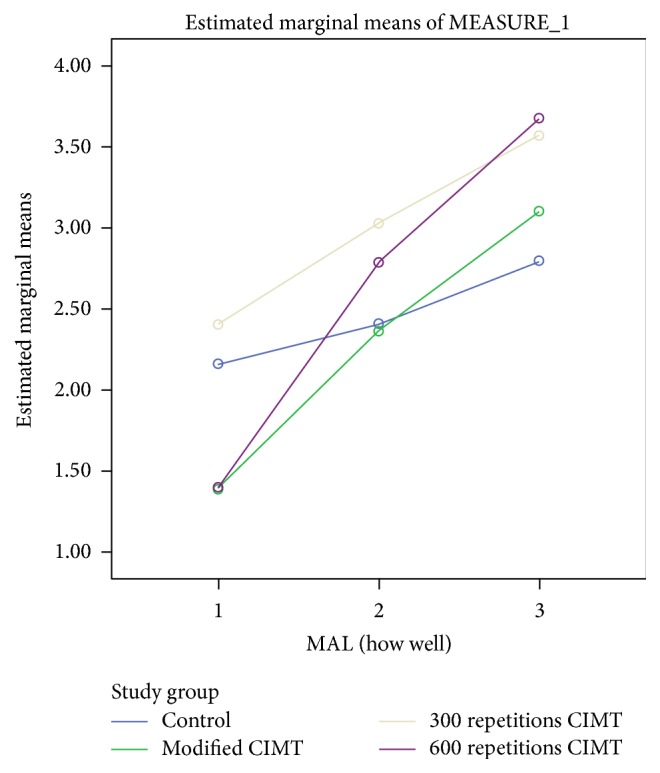
A profile plot showing groups' mean scores in MAL (how well) at baseline, 2 weeks, and 4 weeks after intervention.

For MAL (how well), there was also significant interaction between group and time, Wilks' lambda = .587, *F*(3, 48) = 4.380, *p* = 0.001, and partial *η*^2^ = 0.234. This shows that the changes in MAL (how well) scores over time were not the same between groups. The improvement in MAL (how well) scores attained minimal clinically important difference (MCID) between baseline and 2 weeks in only 600 repetitions group and between baseline and 4 weeks after intervention in only mCIMT, 300 repetitions, and 600 repetitions groups (see Table 2 and Figure 3). The MCIDs for MAL (how well) for dominant and nondominant hands are 1.0 and 1.1 points, respectively [30]. However, there was a substantial main effect for time, Wilks' lambda = 0.183, *F*(3, 48) = 95.980, *p* < 0.001, and partial *η*^2^ = 0.817, with all groups showing increase in MAL (how well) scores across the three time periods (see Figure 3). The main effect comparing the 3 groups was not significant, *F*(3, 48) = 0.890, *p* = 0.057, and partial *η*^2^ = 0.454, suggesting no difference in the effectiveness of the four interventions.

**Figure 4:**
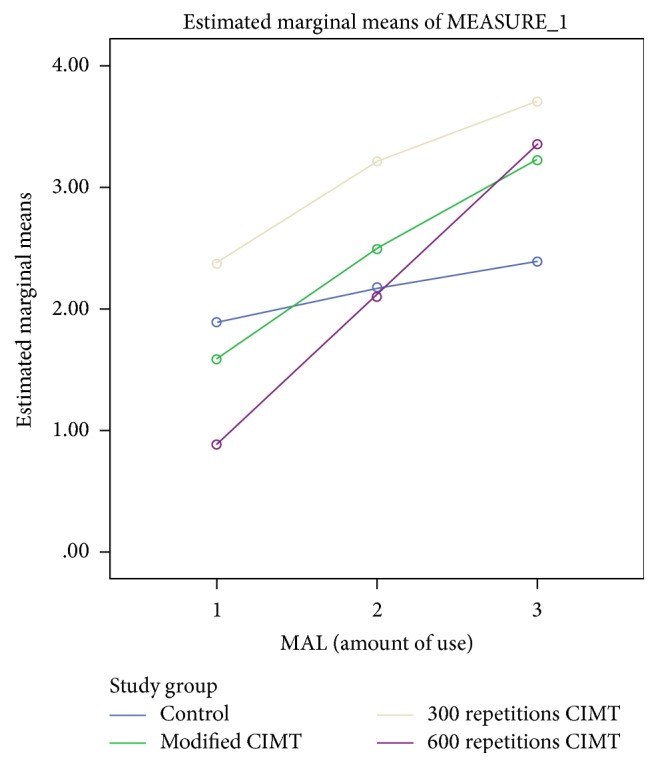
A profile plot showing groups' mean scores in MAL (amount of use) at baseline, 2 weeks, and 4 weeks after intervention.

For MAL amount of use, there was also significant interaction between group and time, Wilks' lambda = .447, *F*(3, 48) = 7.107, *p* < 0.001, and partial *η*^2^ = 0.331. This shows that the changes in MAL (amount of use) scores over time were not the same between groups. At baseline, control and 300 repetitions groups had higher mean scores; at 2 weeks after intervention, mCIMT and 300 repetitions groups had higher mean scores; and at 4 weeks after intervention, 600 repetitions and 300 repetitions groups had higher mean scores (see Table 2 and Figure 4). However, there was a substantial main effect for time, Wilks' lambda = 0.156, *F*(3, 48) = 115.992, *p* < 0.001, and partial *η*^2^ = 0.844, with all groups showing increase in MAL (amount of use) scores across the three time periods (see Figure 4 and Table 2). The main effect comparing the 3 groups was not significant (*F*(3, 48) = 2.093, *p* = 0.115, and partial *η*^2^ = 0.125), suggesting no difference in the effectiveness of the four interventions.

**Figure 5:**
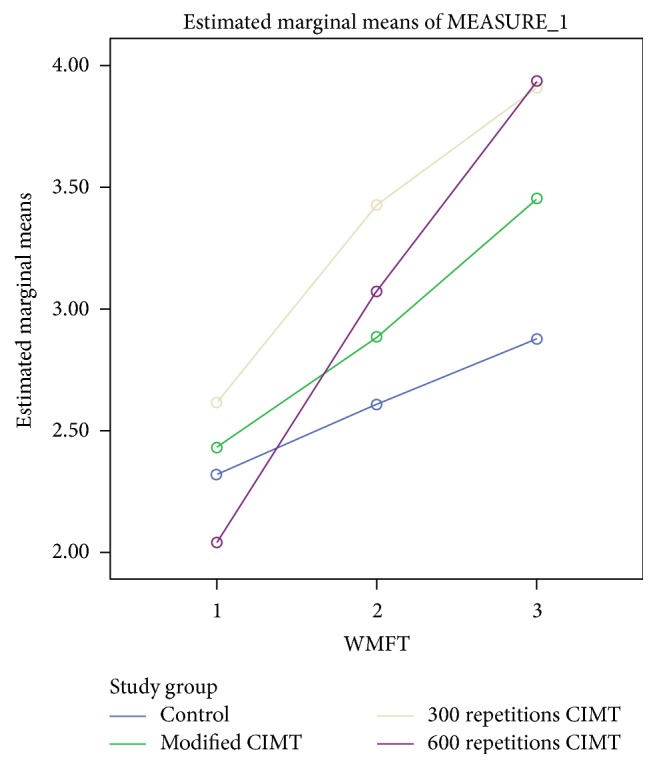
A profile plot showing groups' mean scores in WMFT at baseline, 2 weeks, and 4 weeks after intervention.

For WMFT, there was significant interaction between group and time, Wilks' lambda = .608, *F*(3, 48) = 4.046, *p* = 0.001, and partial *η*^2^ = 0.220. This shows that the changes in WMFT scores over time were not the same between groups. The improvement in WMFT scores attained minimal clinically important difference (MCID) between baseline and 4 weeks after intervention in only mCIMT, 300 repetitions, and 600 repetitions groups (see Table 2 and Figure 5). The MCIDs for WMFT functional activities for dominant and nondominant hands are 1.0 and 1.2 points, respectively [30]. However, there was a substantial main effect for time, Wilks' lambda = 0.209, *F*(3, 48) = 81.213, *p* < 0.001, and partial *η*^2^ = 0.791, with all groups showing increase in WMFT scores across the three time periods (see Figure 5). The main effect comparing the 3 groups was not significant (*F*(3, 48) = 1.391, *p* = 0.258, and partial *η*^2^ = 0.087), suggesting no difference in the effectiveness of the four interventions.

**Figure 6:**
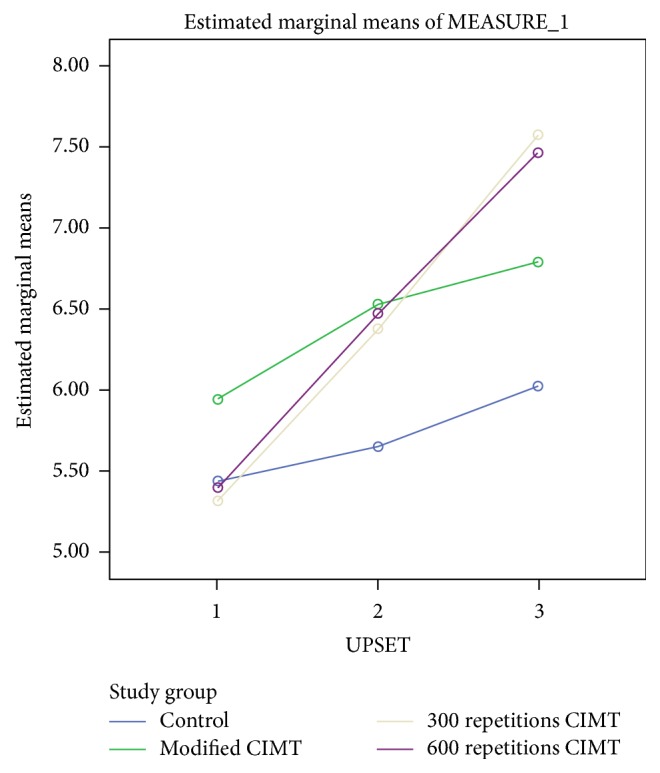
A profile plot showing groups' mean scores in UPSET at baseline, 2 weeks, and 4 weeks after intervention.

For UPSET, there was significant interaction between group and time, Wilks' lambda = .719, *F*(3, 48) = 2.574, *p* = 0.024, and partial *η*^2^ = 0.152. This shows that the changes in UPSET scores over time were not the same between groups. At baseline, mCIMT group had higher mean scores; at 2 weeks after intervention, mCIMT and 600 repetitions groups had higher mean scores; and at 4 weeks after intervention, 600 repetitions and 300 repetitions groups had higher mean scores (see Table 2 and Figure 6). However, there was a substantial main effect for time, Wilks' lambda = 0.332, *F*(3, 48) = 43.229, *p* < 0.001, and partial *η*^2^ = 0.668, with all groups showing increase in UPSET scores across the three time periods (see Table 2). The main effect comparing the 3 groups was not significant (*F*(3, 48) = 0.686, *p* = 0.565, and partial *η*^2^ = 0.045), suggesting no difference in the effectiveness of the four interventions.

## 4. Discussion

The study was aimed at comparing the use of number of repetitions of shaping practice and the number of hours of shaping practice during CIMT. The results of the study showed that there was no significant difference between groups in the outcomes of interest. However, the within-group improvement in the outcomes of interest attained minimal clinically important difference (MCID) in modified CIMT, 300 repetitions, and 600 repetitions groups. The MCID is “the smallest difference in score in the domain of interest which patients perceive as beneficial and which would mandate, in the absence of troublesome side effects and excessive cost, a change in the patient's management [31].”

The improvements across time periods in motor function, real-world arm use, and self-efficacy seen in modified CIMT, 300 repetitions, and 600 repetitions groups, respectively, are encouraging. This is because CIMT studies had previously reported improvements in motor function and real-world arm use [10, 32, 33]. The studies, however, used number of hours of shaping/tasks practice, a protocol that has been questioned not to clearly show how much tasks/shaping is being practiced [13, 14]. Additionally, most of the laboratory protocols are personnel- and time-intensive and require supervision of dedicated therapists [8]. Consequently, efforts were made to relieve personnel using a computerized mechanical device known as AUTOCITE [34, 35]. Although the effort is very laudable, it did not completely address the issues surrounding task/shaping practice during CIMT. For instance, patients will have to still spend several or many hours practicing, even though whether they actually and completely used the hours for practice may not be accounted for [9, 10].

The protocol of the present study in contrast to the one using number of hours clearly states the number of repetitions required. Such a protocol is simple and can easily be monitored even by the patients themselves [36]. Additionally, the protocol is a sort of self-management, and this will empower patients to take charge of their own recovery process and free some time for the therapists. However, the study has some limitations such as inability to obtain the required sample size calculated for the study. Inadequate sample size could underestimate differences between groups, though a sample size should be neither small nor excessive [37]. Another limitation of the study was that number of repetitions in the group that used hours of practice and number of hours or time spent in the group that used number of repetitions were not estimated.

## 5. Conclusion

Number of repetitions of shaping practice improved motor function, real-world arm use, and self-efficacy. Although these improvements did not differ significantly from those produced by modified CIMT, there is indication that the use of number of repetitions of shaping practice may effectively serve as a simple and clear alternative to the protocol using number of hours of shaping practice. However, further studies are needed to determine the mean time during which patients can perform 300 or 600 repetitions of shaping practice.

## Figures and Tables

**Table 1:** Baseline characteristics of the study participants.

Variable	Group				Statistics	*p*
	Control (*n* = 12)	Modified CIMT (*n* = 13)	300 repetitions (*n* = 12)	600 repetitions (*n* = 11)		
Age	58.83 ± 10.57	54.62 ± 6.00	59.42 ± 13.93	57.60 ± 10.27		
Time since stroke	19.89 ± 7.20	14.75 ± 4.46	21.67 ± 6.38	13.50 ± 7.39		
Sex	20.00	21.38	30.00	27.09		
Type of stroke	26.50	24.04	20.50	27.23		
Hand dominance	23.00	24.85	25.00	25.18		
Side affected	26.00	23.38	26.00	22.55		
MAL (how well)	2.13 ± 1.32	1.62 ± 0.73	2.30 ± 1.16	1.51 ± 0.94	*F* = 1.577	0.208
MAL (amount of use)	1.91 ± 1.37	1.75 ± 0.54	2.31 ± 1.19	1.18 ± 0.77	*F* = 2.433	0.078
WMFT	2.22 ± 1.19	2.17 ± 0.82	1.05 ± 0.30	1.99 ± 0.52	*F* = 0.524	0.668
FM	31.42 ± 16.21	34.00 ± 14.15	35.17 ± 13.02	30.45 ± 10.76	*F* = 0.299	0.826
UPSET	4.75 ± 3.26	4.58 ± 2.21	4.74 ± 1.88	4.56 ± 1.81	*F* = 0.021	0.996

**Table 2:** Groups' mean scores at baseline, 2 weeks, and 4 weeks after intervention in the outcomes of interests.

Variable	Group	*n*	Baseline	2 weeks	4 weeks	Within-group mean difference		
						B to 2	2 to 4	B to 4
FM	Control	12	32.73 ± 16.32	35.09 ± 16.56	35.90 ± 16.48	2.36	0.81	3.17
	Modified CIMT	13	34.00 ± 14.15	44.69 ± 9.98	50.69 ± 8.60	10.69^*∗*^	6.00^*∗*^	16.69^*∗*^
	300 repetitions	12	35.17 ± 13.02	45.33 ± 10.89	52.50 ± 8.54	10.16^*∗*^	7.17^*∗*^	17.33^*∗*^
	600 repetitions	11	30.45 ± 10.76	41.55 ± 7.99	49.27 ± 6.23	11.10^*∗*^	7.72^*∗*^	18.82^*∗*^

MAL (how well)	Control	12	2.13 ± 1.32	2.39 ± 1.28	2.70 ± 1.07	0.26	0.31	0.57
	Modified CIMT	13	1.62 ± 0.73	2.48 ± 0.77	3.25 ± 0.78	0.86	0.77	1.63^*∗*^
	300 repetitions	12	2.30 ± 1.16	2.98 ± 0.96	3.57 ± 0.90	0.68	0.59	1.27^*∗*^
	600 repetitions	11	1.51 ± 0.94	2.69 ± 0.79	3.47 ± 0.64	1.18^*∗*^	0.78	1.96^*∗*^

MAL (AOU)	Control	12	1.91 ± 1.37	2.16 ± 1.30	2.36 ± 1.12			
	Modified CIMT	13	1.75 ± 0.54	2.60 ± 0.76	3.40 ± 0.84			
	300 repetitions	12	2.31 ± 1.19	3.07 ± 1.15	3.56 ± 0.87			
	600 repetitions	11	1.18 ± 0.77	2.18 ± 0.47	3.32 ± 0.42			

WMFT	Control	12	2.22 ± 1.19	2.47 ± 1.06	2.67 ± 1.00	0.25	0.20	0.45
	Modified CIMT	13	2.17 ± 0.82	2.68 ± 0.81	3.40 ± 0.82	0.51	0.72	1.23^*∗*^
	300 repetitions	12	2.47 ± 1.20	3.19 ± 1.16	3.79 ± 0.92	0.72	0.60	1.62^*∗*^
	600 repetitions	11	1.99 ± 0.52	2.92 ± 0.47	3.71 ± 0.54	0.93	0.79	1.72^*∗*^

UPSET	Control	12	4.75 ± 3.26	5.07 ± 3.11	5.43 ± 3.05			
	Modified CIMT	13	4.58 ± 2.21	6.02 ± 1.57	7.10 ± 1.42			
	300 repetitions	12	4.74 ± 1.88	6.12 ± 1.79	7.68 ± 1.89			
	600 repetitions	11	4.56 ± 1.81	6.16 ± 1.33	7.34 ± 1.21			

*∗* indicates attained minimal clinically important difference (MCID). B to 2: baseline to 2 weeks; 2 to 4: 2 weeks to 4 weeks; B to 4: baseline to 4 weeks.

## Data Availability

The data is available on request from the author.
