# Corrigendum to “Effects of Number of Repetitions and Number of Hours of Shaping Practice during Constraint-Induced Movement Therapy: A Randomized Controlled Trial”

**DOI:** 10.1155/2020/1256231

**Published:** 2020-08-24

**Authors:** Auwal Abdullahi

**Affiliations:** Department of Physiotherapy, Bayero University Kano, Kano, Kano State, Nigeria

In the article titled “Effects of Number of Repetitions and Number of Hours of Shaping Practice during Constraint-Induced Movement Therapy: A Randomized Controlled Trial” [1], there was an error in Table 1 in which the incorrect data were given for sex, type of stroke, hand dominance, and side affected. The author apologises for this error and the corrected table is shown below.

## Figures and Tables

**Table 1:** Baseline characteristics of the study participants.

Variable	Control (*n* = 12)	Group			Statistics	*p*
		Modified CIMT (*n* = 13)	300 Rep (*n* = 12)	600 Rep (*n* = 11)		
Age	58.83 ± 10.57	54.62 ± 6.00	59.42 ± 13.93	57.60 ± 10.27		
Time since stroke	19.89 ± 7.20	14.75 ± 4.46	21.67 ± 6.38	13.50 ± 7.39		
Sex (M/F)	9/3	9/4	4/8	5/6		
Type of stroke (I/H)	8/4	10/3	11/1	7/4		
Hand dominance (R/L)	12/0	12/1	11/1	10/1		
Side affected (R/L)	7/5	9/4	7/5	8/3		
MAL (how well)	2.13 ± 1.32	1.62 ± 0.73	2.30 ± 1.16	1.51 ± 0.94	*F* = 1.577	0.208
MAL (amount of use)	1.91 ± 1.37	1.75 ± 0.54	2.31 ± 1.19	1.18 ± 0.77	*F* = 2.433	0.078
WMFT	2.22 ± 1.19	2.17 ± 0.82	1.05 ± 0.30	1.99 ± 0.52	*F* = 0.524	0.668
FM	31.42 ± 16.21	34.00 ± 14.15	35.17 ± 13.02	30.45 ± 10.76	*F* = 0.299	0.826
UPSET	4.75 ± 3.26	4.58 ± 2.21	4.74 ± 1.88	4.56 ± 1.81	*F* = 0.021	0.996

R/L = right/left; I/H = ischaemic/haemorrhagic; M/F = male/female.
